# Biofabrication of *in situ* Self Assembled 3D Cell Cultures in a Weightlessness Environment Generated using Magnetic Levitation

**DOI:** 10.1038/s41598-018-25718-9

**Published:** 2018-05-08

**Authors:** Muge Anil-Inevi, Sena Yaman, Ahu Arslan Yildiz, Gulistan Mese, Ozden Yalcin-Ozuysal, H. Cumhur Tekin, Engin Ozcivici

**Affiliations:** 1 Department of Bioengineering, Izmir Institute of Technology, Urla, Izmir Turkey; 2 Department of Molecular Biology and Genetics, Izmir Institute of Technology, Urla, Izmir Turkey

## Abstract

Magnetic levitation though negative magnetophoresis is a novel technology to simulate weightlessness and has recently found applications in material and biological sciences. Yet little is known about the ability of the magnetic levitation system to facilitate biofabrication of *in situ* three dimensional (3D) cellular structures. Here, we optimized a magnetic levitation though negative magnetophoresis protocol appropriate for long term levitated cell culture and developed an *in situ* 3D cellular assembly model with controlled cluster size and cellular pattern under simulated weightlessness. The developed strategy outlines a potential basis for the study of weightlessness on 3D living structures and with the opportunity for real-time imaging that is not possible with current ground-based simulated weightlessness techniques. The low-cost technique presented here may offer a wide range of biomedical applications in several research fields, including mechanobiology, drug discovery and developmental biology.

## Introduction

Cells in living organisms are continuously exposed to varying degrees of mechanical forces, which serve as critical stimuli and influence their fate^1–4^. Such physical signals are key regulators of organ system maintenance, repair and renewal in mammals^5,6^. Permanent or temporary reduction of mechanical stimulations, as experienced during spaceflight, immobilization, paralysis and bed rest, cause deteriorations in the human body^7^, especially in the musculoskeletal system such as demineralization of bones and mass loss of skeletal muscle^8–12^. Spaceflight experiments offer great opportunities to improve our understanding on short term and long duration biological effects of weightlessness^13–15^. Nevertheless, such experiments are rare, expensive to operate and hard to secure, and alternative ground-based techniques have hence been developed to simulate the weightlessness environment^16^. The most commonly used devices to study simulated weightlessness are the rotating-wall vessel (RWV) platform^17–19^, 2D clinostats^20–22^ and Random Positioning Machines (RPM)^20,23,24^. However, these devices create fluid shear stress on the cells due to rotation and this can interrupt the response of cells to a randomized gravity vector^25,26^. Furthermore, both the clinostat and the RPM requires time for randomization of gravity vector and therefore they are not convenient for relatively rapidly occurring cellular processes.

One of the most recent ground based technology to mimic the biological effects of weightlessness is magnetic levitation technique^27^. Magnetic levitation can be applied via positive or negative magnetophoresis, however positive magnetophoresis (i.e. magnetic bead labeling technique) cannot simulate weightlessness because acting forces that levitate the subject of interest only act on the surface of the subject and any internal structures are free of those forces^28,29^. In contrast, levitation through negative magnetophoresis (also referred to as diamagnetophoresis) can exactly mimic weightlessness. During negative magnetophoresis, gravitational force on the subject is compensated by a counteracting force that induces weightlessness. In contrast to other ground-based methods, magnetic levitation allows the investigation of relatively fast cellular processes. In this technique, diamagnetic objects (i.e. almost all cells) are guided towards regions of low magnetic field in a magnetic field gradient and the process is resulted in stable magnetic levitation and the simulation of weightlessness environment as long as the gradient is intact^30–32^. Such a strategy requires high magnitude magnetic fields that can be detrimental to biological subjects^33^. In order to reduce the magnitude of magnetic fields, it is possible to increase the magnetic susceptibility of medium by using paramagnetic solutions^34–36^ or ferrofluids^37^. Recently an inexpensive strategy has been demonstrated for label-free cell levitation in gadolinium (Gd^3+^) based solution^38^ and successfully applied for detection of differences in cell densities at the single-cell level^39^ and guided assembly of *ex situ* generated spheroids^40^. However, self-guided assembly of cells *in situ* during levitation and appropriate Gd^3+^ based solution for longer term culturing is largely unknown.

In this study, we used a magnetic levitation system for *in situ* cell culture in simulated microgravity. First, we investigated the most appropriate composition and concentration for Gd^3+^ based solution for weightlessness culturing. Further, we documented the self-assembly pattern of cells and controlling of cluster size with initial cell number. Finally, we applied our previous findings to determine the possibility of coculture and biofabrication of novel cellular patterns. Our study established the possibility of levitation through diamagnetophoresis as a powerful biomedical tool that will allow testing of molecular and cellular level hypotheses on biological effects of weightlessness in a single cell level that is not possible with current methods simulating weightlessness.

## Results

### Short-term levitation of cells with different Gd-based solutions

**Figure 1:**
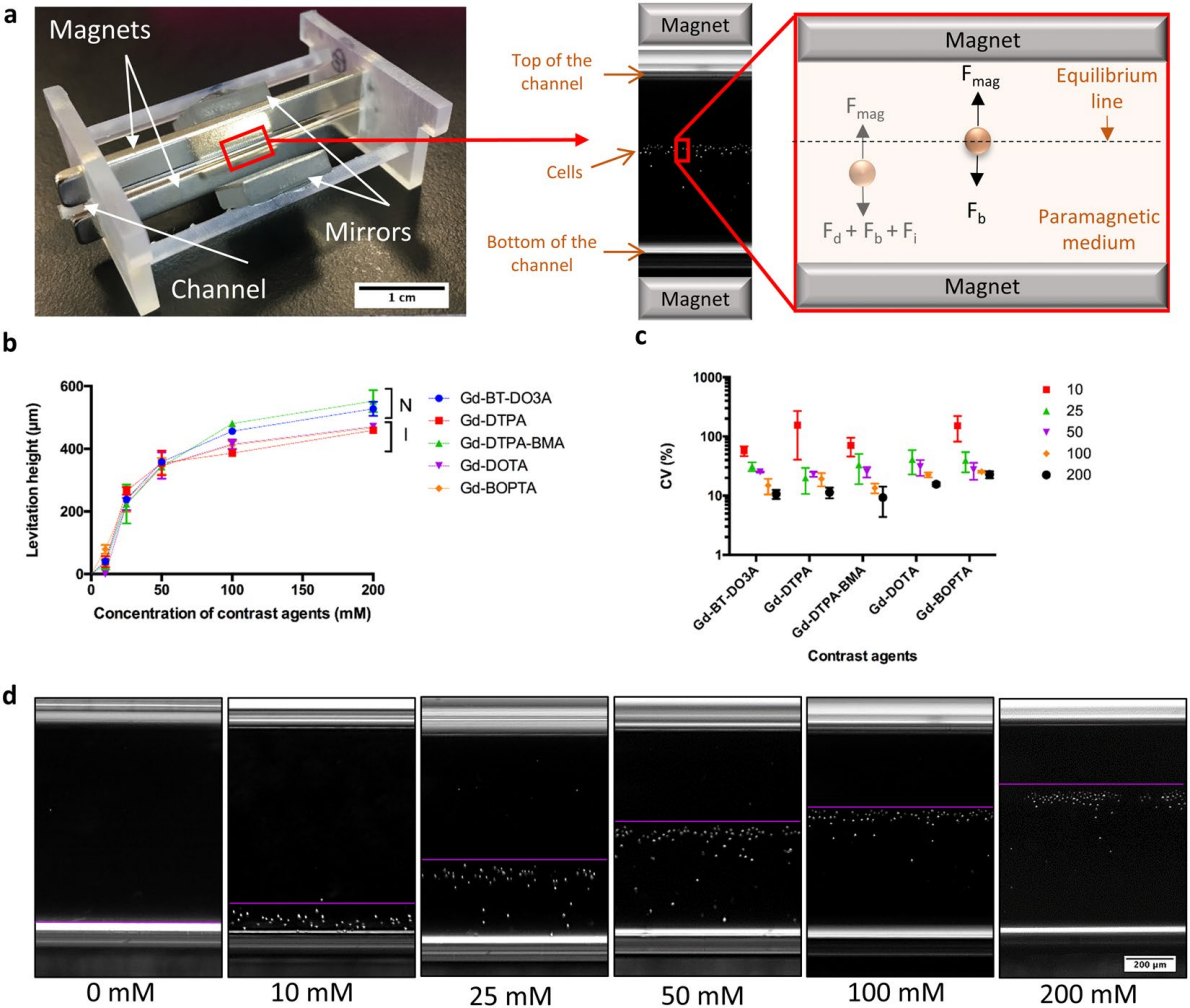
Short-term levitation of D1 ORL UVA cells in the magnetic levitation platform. (**a**) Photograph of magnetic levitation platform and forces which act on cells in it until equilibrium; fluidic drag force (F_d_), inertial force (F_i_), buoyancy force (F_b_) and magnetic force, F_mag_, and at the equilibrium position; F_mag_ and F_b_. Microcapillary channel, in which cells are levitated and cultured, is placed between two permanent neodymium magnets whose same negative poles are facing each other. Mirrors are placed at each open side of the channel at 45° and used to visualize cells in the channel with conventional microscopy systems. (**b**,**c**) The relationship between Gd concentrations and, levitation heights of cells (from bottom surface of capillary) (**b**) and CV (%) of levitation heights (**c**) after 10 min of levitation in different Gd-based solutions (Gd-BT-DO3A, Gd-DTPA, Gd-DTPA-BMA, Gd-DOTA and Gd-BOPTA). Data are plotted as mean of replicates with error bars (±SD). N: nonionic agents, I: ionic agents. (**d**) Micrographs of levitated cells after 10 min of levitation in the medium containing Gd-BT-DO3A at variable concentrations (0, 10, 25, 50, 100 and 200 mM). The lines show the upper level of the levitated cell population. Scale bar: 200 μm.

In order to select the most appropriate media for cell culture during magnetic levitation, we used a custom made microfluidic levitation device (Fig. 1a, Supplementary Information, Supplementary Fig. 1) to levitate D1 ORL UVA bone marrow mesenchymal stem cells with different Gd-based contrast agents; gadobutrol (Gd-BT-DO3A), gadopentetate dimeglumine (Gd-DTPA), gadodiamide (Gd-DTPA-BMA), gadoterate meglumine (Gd-DOTA) and gadobenate dimeglumine (Gd-BOPTA) at increasing concentrations (0, 10, 25, 50, 100 and 200 mM) and measured location of cells from bottom surface of capillary after 10 min of levitation to allow cells levitated at lower concentrations of Gd^3+^ to reach steady state (Fig. 1a,b,d and Supplementary Fig. 2). Irrespective of the chemical composition of the Gd-based agent, increasing concentrations resulted in increased levitation height of cells. Levitation heights of cells at concentration of 100 mM solutions reached more than 80% of the cell heights observed at 200 mM concentrations (86.4, 84.1, 87.1, 88.1 and 88.1% for Gd-BT-DO3A, Gd-DTPA, Gd-DTPA-BMA, Gd-DOTA and Gd-BOPTA, respectively). Furthermore, nonionic structure containing Gd-BT-DO3A and Gd-DTPA-BMA, provided higher levitation heights at Gd^3+^ concentrations of 100 and 200 mM than ionic structure containing ones (Gd-DTPA, Gd-DOTA and Gd-BOPTA). Levitation heights for non-ionic structure containing solutions were 15.87% (p < 0.0001) and 15.95% (p < 0.0001) higher compared to ionic structure containing solutions at 100 mM and 200 mM concentrations (Fig. 1b). According to the coefficient of variation (CV%) of calculated cellular levitation (Fig. 1c), increasing concentrations not only reduced CV% values, but also standard deviation of CV% values as well, showing a reduction in variability within- and between-experiments with increased concentrations. Furthermore, increased Gd concentrations allowed the cells to equilibrate faster in the levitation platform for all agents. All contrast agents, except for Gd-DOTA (equilibrium time: 3 min), immediately provided equilibrium of levitated cells at 200 mM. However, time of equilibrium was higher than 4 min at the concentration of 10 mM Gd^3+^ for all agents (Supplementary Fig. 3a–e and Video 1).

### Long-Term Culture of Cells in Gd-based solutions

**Figure 2:**
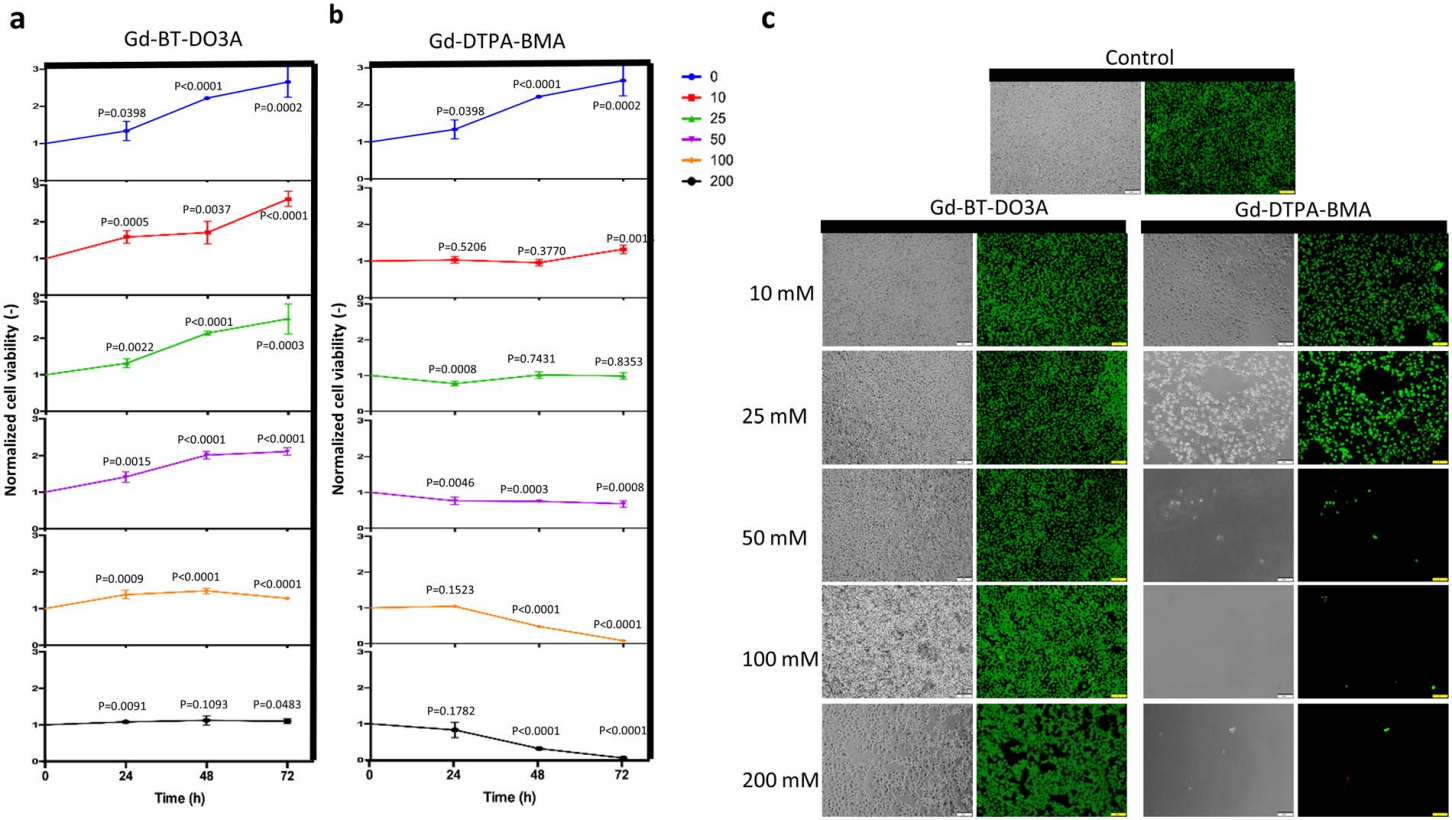
Long-term culture viability results of D1 ORL UVA cells. (**a**,**b**) Cell viability for long-term culturing with Gd-BT-DO3A and Gd-DTPA-BMA at increasing concentrations (0, 10, 25, 50, 100 and 200 mM), respectively. Cell viability was determined with MTT assay. Data are plotted as mean of replicates with error bars (±SD). Groups were evaluated using the unpaired Student’s t-test. Statistical significance was defined as P < 0.05. (**c**) Fluorescent and phase-contrast microscopy images of D1 ORL UVA cells cultured for 72 h with 0, 10, 25, 50, 100 and 200 mM Gd-BT-DO3A and Gd-DTPA-BMA (live: green, dead: red). Cell viability was visualized by live-dead staining (Calcein/PI). Cells were cultured in the standard culture medium, as a control. Scale bars: 100 μm.

To quantify the impact of Gd-based solutions on cell viability for long-term culturing, we cultured D1 ORL UVA cells on plate with different paramagnetic medium at increasing concentrations (0, 10, 25, 50, 100 and 200 mM) and measured cell viability by MTT assay (Fig. 2a,b and Supplementary Fig. 4). Gd-DTPA, Gd-DTPA-BMA, Gd-DOTA and Gd-BOPTA led to massive cell death after 72 h of incubation at 200 mM concentration, whereas, Gd-BT-DO3A only prevented cell growth. For other concentrations, macrocyclic ligand containing Gd-BT-DO3A and Gd-DOTA provided higher cell viability compared to linear ligand containing solutions (Gd-DTPA, Gd-DTPA-BMA and Gd-BOPTA). Cells cultured with macrocyclic ligand containing solutions during 72 h had 70.3% (p < 0.0001), 125.39% (p < 0.0001), 160.25% (p < 0.0001) and 219.94% (p < 0.0001) higher cell viability than cells cultured with linear ligand containing solutions at 10 mM, 25 mM, 50 mM and 100 mM concentrations, respectively. Gd-DTPA-BMA, one of the two contrast agents providing high levels of levitation, inhibited cell growth even at low concentrations (25 mM), while Gd-BT-DO3A, showed a 27.92% increase in cell viability after 72 h of culture at high concentration (100 mM). Besides, the effects of these two contrast agents on cell viability were also assessed by live/dead assay (Fig. 2c). Cells cultured with Gd-BT-DO3A including 100 mM for 72 h exhibited similar viability and confluency compared to the control group. For better display of dead cells, micrographs presented in Fig. 2c were zoomed-in (Supplementary Fig. 5) showing that the ratio of dead cells in these concentrations were comparable with control culture. Consistent with MTT results, at the concentration of 200 mM Gd-BT-DO3A, non-uniform gaps began to form in the culture indicating cell loss. Culture with Gd-DTPA-BMA agent at concentrations of 50 mM and above resulted in almost complete cell loss, and the culture at 25 mM did not only lead to a decrease in cell confluency but also to increase in the size of the cells.

Commercial contrast agents added to the culture medium dilute the medium in varying proportions. Stock concentrations of the agents used in the study were 1000 mM (Gd-BT-DO3A) and 500 mM (Gd-DTPA, Gd-DTPA-BMA, Gd-DOTA and Gd-BOPTA) and for the highest concentration (200 mM), agents constituted 20% and 40% of the medium, respectively. In order to assess whether this product-related difference had an effect on cell viability, D1 ORL UVA cells were cultured in the medium containing PBS, instead of the contrast agent (0, 10, 20, 30, 40 and 50%; v/v) for 24, 48 and 72 h and cell viability was tested by MTT assay (Supplementary Fig. 6). There was no statistically significant difference between the viability of cells grown in 20% and 40% PBS containing medium, which mimics the 200 mM agent concentrations, for 24 (P = 0.27), 48 (0.14) and 72 h (P = 0.08). Taken together, Gd-BT-DO3A was chosen for following levitation experiments due to providing both higher levitation height and at least 43.9% higher cell viability than the other agents after 72 h of culture at all concentrations (except for Gd-DOTA).

### Self-guided 3D cellular assembly during weightlessness

Before levitation of cells at the standard culture conditions (37 °C), D1 ORL UVA^eGFP^ cells were levitated at varying temperatures (28, 32 or 36 °C) with 50, 100, and 200 mM Gd-BT-DO3A solution and the levitation was examined after time of equilibrium (Supplementary Fig. 7). The results showed that cells reached similar equilibrium levels at all tested temperatures and Gd-BT-DO3A concentrations. Following the observation that slight temperature changes had no effect on cell levitation heights, cell culture studies were performed in the levitation device. Cells were first levitated and cultured for 72 h in the levitation system using 50 mM Gd-BT-DO3A concentration, which appeared to be advantageous in terms of cell viability and provided sufficient levitation height. Although the levitated cell spheres were observed after assembly, some cellular clusters collapsed and attached on the ground of the capillaries during culture time (Supplementary Fig. 8). We therefore increased Gd-BT-DO3A concentration to 100 mM for culturing, which was also suitable for cell viability and adequate cell levitation height.

First, to investigate whether 100 mM Gd-BT-DO3A exposure is suitable for longer-term cell culture with regard to cell viability, we exposed D1 ORL UVA cells to 100 mM Gd-BT-DO3A for 120 h in 2D culture (Supplementary Fig. 9a–d) and in 3D magnetic levitation (Supplementary Fig. 9e–j). In 2D culture, cells showed healthy morphology at 24^th^, 72^nd^ and 120^th^ h of exposure to 100 mM Gd-BT-DO3A (Supplementary Fig. 9a–c) and limited number of dead cells were observed after 120 h (Supplementary Fig. 9d). In 3D magnetic levitation, cells maintained their cluster shape at 24^th^, 72^nd^ and 120^th^ h of magnetic levitation (Supplementary Fig. 9e–g) and the clustered cells were viable for 120 h (Supplementary Fig. 9h,i). For a better observation of dead cells within the clusters, we dissociated clusters into single cell suspension with gentle pipetting for live/dead staining and consistently large majority of the cells were found to be viable (Supplementary Fig. 9j).

**Figure 3:**
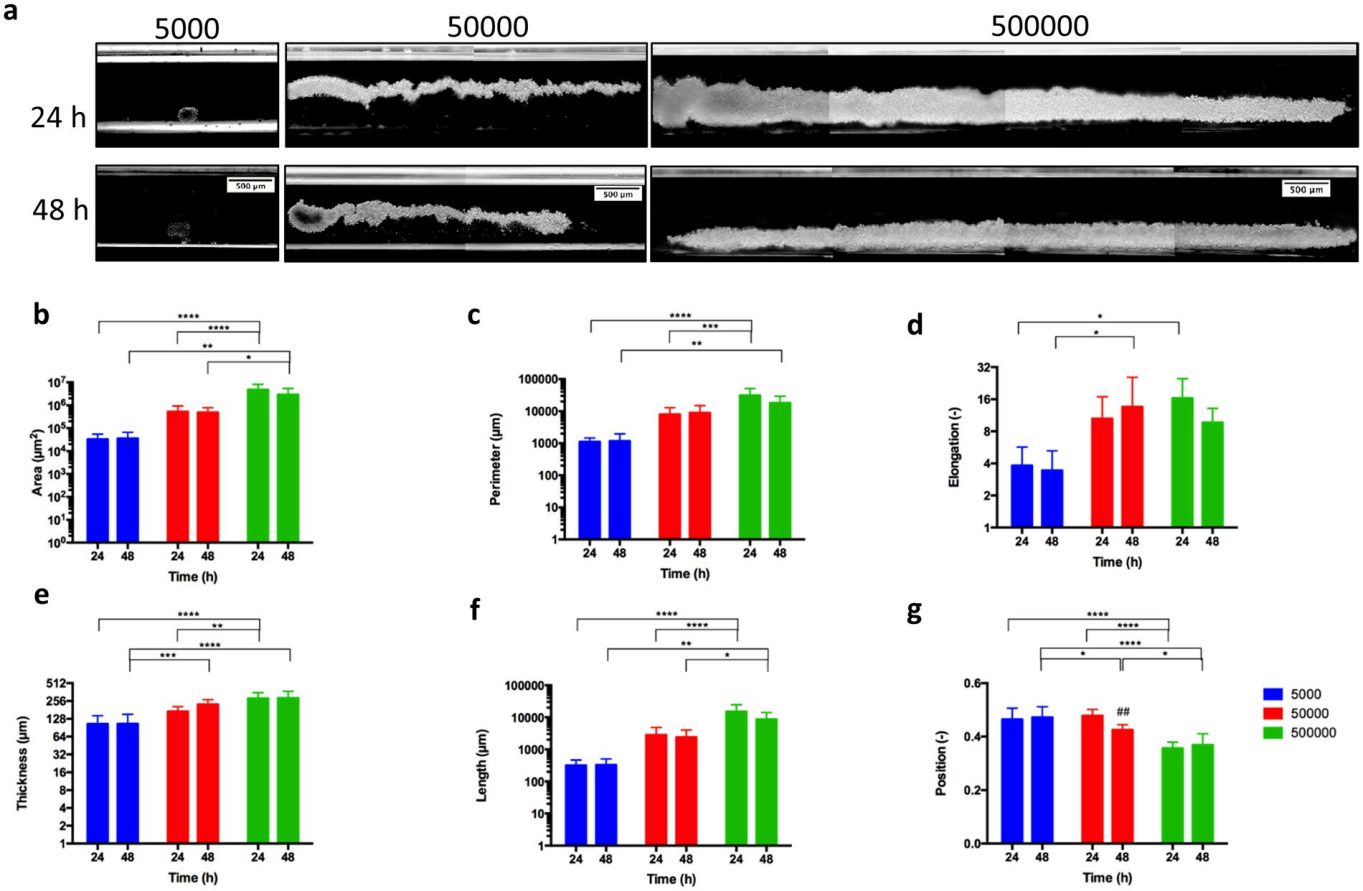
3D cellular organization of D1 ORL UVA cells under microgravity. (**a**) Micrographs of cells levitated and assembled for 24 and 48 h (with 100 mM Gd-BT-DO3A) at different cell numbers (total 5000, 50000 and 500000 cells). Scale bars: 500 μm. (**b**–**f**) Quantitative description of the cellular clusters formed for 24 or 48 h with magnetic levitation (100 mM Gd-BT-DO3A) at different cell numbers (total 5000, 50000 and 500000 cells); (**b**) area, (**c**) perimeter, (**d**) elongation, (**e**) thickness and (**f**) length, and (**g**) position of the clusters between magnets (the top point of the bottom magnet:0, the bottom point of the top magnet: 1). Data are plotted as mean of replicates with error bars (±SD) and statistically analyzed using a two-way ANOVA and Sidak posthoc test. Statistical significance was defined as P < 0.05.

Second, to investigate the effect of cell number increase on the morphology of cell clusters forming under microgravity condition; 5000, 50000 or 50000 D1 ORL UVA cells were levitated at 100 mM Gd-BT-DO3A, cultured in the levitation device for 48 h and resultant cluster morphologies were analyzed and all clusters in each experiment were averaged (Fig. 3). There was no statistical difference between the area, perimeter, elongation, thickness and length of the cellular constructs formed at the 24th hour of the culture, and at the 48th hour of the culture. However, as expected, the increase in the number of cells led to an increase in these shape parameters (Fig. 3a–f). The results showed that, when 500000 cells were seeded, the morphological parameters of cellular constructs at 24^th^ hour increased by 87.6, 16.1, 2.5, 2.8 and 21.1-fold compared to 5000 cells for area, perimeter, elongation, thickness and length, respectively. This increase in the size of the structures occurred in the direction of the length rather than the thickness (9.9-fold higher increase), owing to the tendency of the cells to remain in the low magnetic field. The center of mass of the formed clusters was lowered as the number of cells increased, possibly due to the tighter clusters formed by cells (Fig. 3g). Besides, to understand the formation of the stabilized cell clusters, which were observed after 24 h of the culture, the cultured cells were visualized during 10 h in the levitation device (Supplementary Fig. 10 and Video 2). In the first 5 h of the culture, cells apparently assemble into an unstable thin and long cluster and between 5–10 h they stabilize the structure by the shortening of cluster.

### Biofabrication of biphasic assemblies during weightlessness

**Figure 4:**
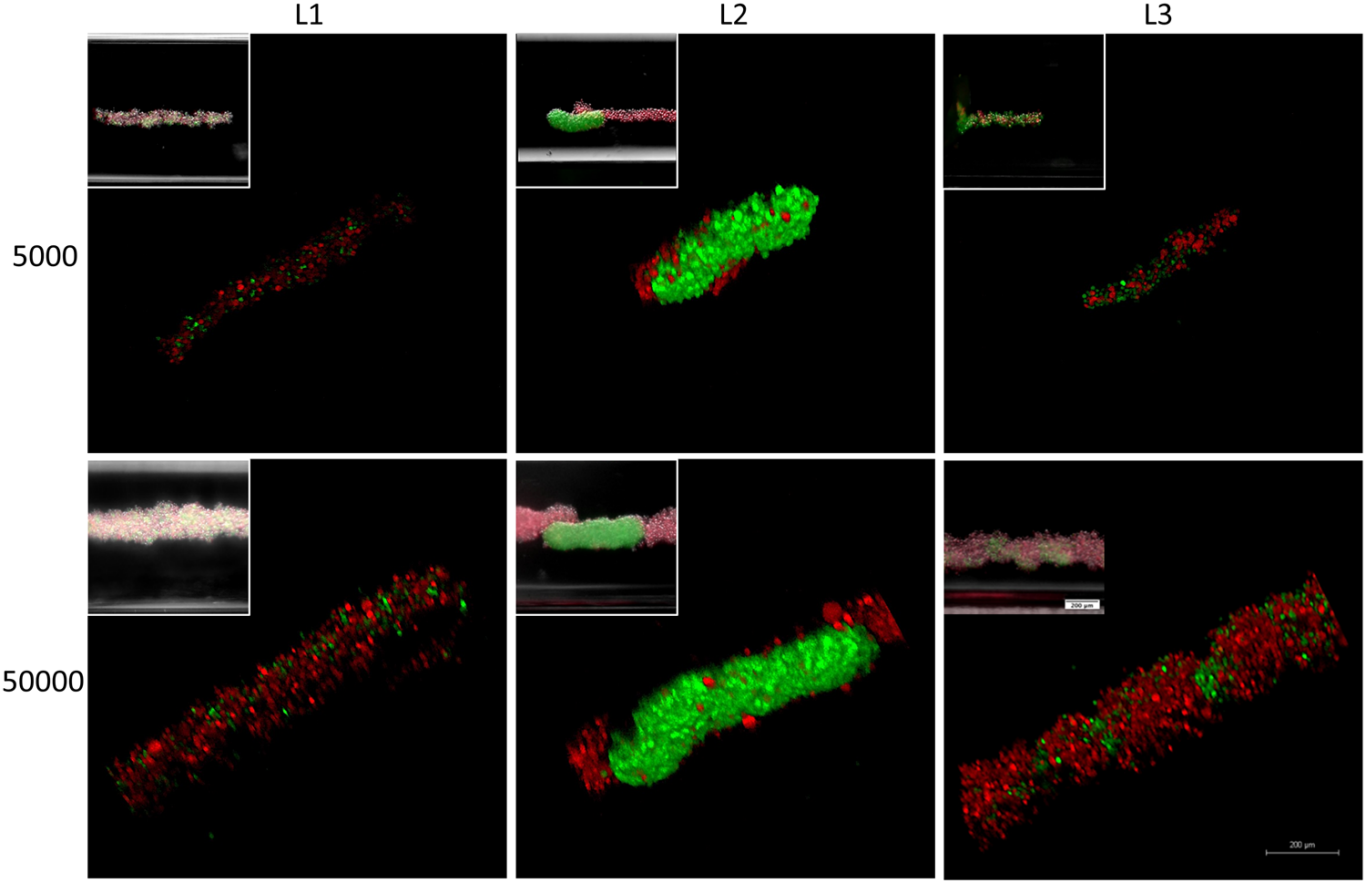
Cellular assembly of D1 ORL UVA^eGFP^ and MDA-MB-231^dsRed^ cells under microgravity. Confocal and conventional fluorescence microscopy (upper left) images showing self-assembled coculture clusters formed with magnetic levitation (100 mM Gd-BT-DO3A) and different cell loading strategies; L1: simultaneously loading of MDA-MB-231^dsRed^ and D1 ORL UVA^eGFP^ cells, L2: MDA-MB-231^dsRed^ cells onto D1 ORL UVA^eGFP^ clusters formed with magnetic levitation and L3: D1 ORL UVA^eGFP^ cells onto MDA-MB-231^dsRed^ clusters formed with magnetic levitation (total 5000 or 50000 cells). Scale bars: 200 μm.

To examine self-assembly of different cell types with different cell to cell adhesion characteristics during weightlessness and to form multiple assembly models with magnetic levitation, D1 ORL UVA^eGFP^ cells (bone marrow stem cell line), observed to be tightly packed in clusters in this study, were cultured with MDA-MB-231^dsRed^ cells (breast cancer cell line), forming only loose clusters^41^. Before assembly of the coculture, separate levitation characteristics height, assembly and morphology were observed for D1 ORL UVA^eGFP^ and MDA-MB-231^dsRed^ cells induced with 100 mM Gd-BT-DO3A (Supplementary Fig. 11a,b). Levitation heights of D1 ORL UVA^eGFP^ and MDA-MB-231^dsRed^ cells were similar (P = 0.9345). During culturing, MDA-MB-231^dsRed^ cells similarly reached to stable morphology and position in 24 h (Supplementary Fig. 11c–i). However, compared to D1 cells MDA-MB-231 clusters assembled at 24 h of the levitation culture had higher area, perimeter, elongation and length values (4.5, 4.6, 4.5 and 4.8-fold, respectively), suggesting loosely formed structures. After examining separate assembly characteristics of both cell groups during weightlessness, biofabrication of biphasic assemblies was performed using different cell loading strategies with low and high cell density; **L1** (simultaneously loading of MDA-MB-231^dsRed^ and D1 ORL UVA^eGFP^ cells), **L2** (MDA-MB-231^dsRed^ cells onto D1 ORL UVA^eGFP^ self-assembled cluster) and **L3** (D1 ORL UVA^eGFP^ cells onto MDA-MB-231^dsRed^ clusters) (Fig. 4). L1 assembly strategy produced assembled clusters with completely random positioning of cells (Supplementary Fig. 12). L2 loading strategy on the other hand resulted in sputtering of loose MDA-MB-231^dsRed^ cells on the surface of tightly formed D1 ORL UVA^eGFP^ cell clusters during weightlessness (Supplementary Fig. 13). Finally, L3 loading strategy caused periodic patterns showing D1 ORL UVA^eGFP^ integrating themselves into gaps within MDA-MB-231^dsRed^ clusters (Supplementary Fig. 14).

## Discussion

We optimized a cellular magnetic levitation protocol by negative magnetophoresis suitable for long term cell culture and developed an *in situ* self-guided cellular assembly model during weightlessness. In summary, we first sought to determine commercially available chelate form and concentration of gadolinium that would be more appropriate to levitate and culture cells by considering viability and levitation position of the cells in the magnetic levitation system. Second, following the specification of the levitation protocol, we showed cellular dynamics and morphology of *in situ* assembly with varying cell numbers in the short and long term. Lastly, we directed biofabrication of various biphasic cellular organizations formed by coculturing cancer cells and stem cells in the levitation system. Provided that the Gd based magnetizing agent is quite stable^42^ *in vitro* conditions, this strategy may be useful tool to study cellular and molecular level effects of microgravity on various models for future research.

Mechanical manipulation of cells in suspended culture via magnetic forces is relatively common in literature. By using magnetic beads, it is possible to manipulate cells through positive magnetophoresis^43,44^. Cellular manipulation with positive magnetophoresis involves labeling cells with magnetic particles and then magnetizing these particles by applying an external magnetic field. This technology enables the assembly of cells into 3D clusters scaffold free^45^, and cells can even be organized into structures with a desired cellular pattern^46^. This approach is also useful in mechanobiology as it potentiates external application of mechanical forces on 3D cellular clusters in compressive^47^ and tensile^48^ modes. It is entirely possible to attach magnetic nanoparticles to extracellular area of the membrane to avoid toxicity^49^. However, magnetic levitation methodologies using positive magnetophoresis are not suitable to simulate exact weightlessness because of the heterogeneous force distribution on cellular structures. In contrast to these studies our aims to induce weightlessness on cells take advantage of the fact that cells and cellular sub-structures are diamagnetic in nature.

Diamagnetic properties of cells facilitate a repulsion by a force in the opposite direction of the applied magnetic field. This diamagnetic repulsive force on cells can be enhanced by suspending cells in a paramagnetic medium for a noticeable effect^50,51^. In this study, we cultured cells in aqueous solutions containing Gd^3+^ with magnetic field gradient to create microgravity condition. However, free form of Gd^3+^ is toxic due to their size similar to Ca^2+^ which lead to competitive inhibition of cellular processes involving Ca^2+^ ^52^. There are various commercially available forms of Gd^3+^ chelated with a ligand (linear or macrocyclic) to prevent direct toxicity of free ion. These contrast agents can be ionic or nonionic. We evaluated five different chelated form of gadolinium with regard to viability and levitation heights of cells. The results indicated that macrocyclic ligand containing agents (Gd-BT-DO3A and Gd-DOTA) provided higher cell viability compared to linear ligand containing ones, possibly due to their lower dissociation constants and higher chemical stability^42,53^. Although the optimal chemical composition and concentration of Gd^3+^ have been established with regard to cell viability in this study, the presence of various cellular effects of Gd^3+^ depending on the concentration of Gd^3+^ and type of target cells^54,55^ appears to be a limitation of this strategy. Furthermore, we showed that Gd-based agents which do not dissociate into charged particles in solution caused higher levitation of cells and identified most appropriate paramagnetic medium composition to levitate D1 ORL UVA cells during long term culture. The Gd-BT-DO3A, determined to be most appropriate for magnetic levitation and long-term culture of cells in this study, has also been preferred to magnetically manipulate cells in the other studies^39,40,56^. We have validated the method here by testing various chemical composition and concentration of Gd^3+^. Another limitation to be taken into consideration is that the level of specific levitation to which cells are gathered may vary greatly for different cell types with different densities and the magnetic levitation strategy may not be suitable for each cell type combination when producing clusters comprising more than one cell type.

The principle of levitation through diamagnetophoresis with the use of Gd-based solutions, has previously been used for manipulation of cells^39,40^. However, there is still an unmet need for the effect of Gd-based compositions on cell viability and levitation for long term culture. The effects of chemical composition and concentration of gadolinium ion-containing agents on cell viability and the levitation process, which have been investigated in the first part of this study, will be quite useful for further studies using the principle of levitation through diamagnetophoresis with paramagnetic solutions. Moreover, even though magnetic levitation principle has been used previously for the organization of suspended cells and previously clustered cellular blocks via a conventional method, separately^40^, for the first time we showed that large cellular blocks (up to ≈2.68 cm in length) could be formed via magnetic levitation, and that cells were assembled to form first single cell type clusters and then to form coculture clusters in a single magnetic levitation device. Taken together, in this study a diamagnetic levitation strategy is presented as a fast and convenient method to simulate the microgravity condition and to examine its effects on 3D complex cellular organizations formed in the same device. The ground-based simulated microgravity strategy presents several advantages, such as: (1) more cost effective than other ground based systems; (2) suitable for real-time imaging; (3) controllable cellular organization by changing magnetic field gradient pattern; (4) non-toxic to cells; and (5) easy to setup and use. The magnetic levitation-based multi-type cellular assembly strategy established here may allow for a wide range of biomedical studies that is not possible with space flight or other ground based methodologies. This system is also suitable for automatization and operation-specific modification to be applied in several gravitational biology researches, particularly in mechanobiology.

## Methods

### Experimental Setup

Cells suspended in the paramagnetic medium (i.e., Gd-based solution) move away from high magnetic field (i.e. regions close to the magnets) to low magnetic field due to the difference between the magnetic susceptibility of cells and surrounding paramagnetic medium. Until cells reach equilibrium position, fluidic drag (F_d_), inertial (F_i_), buoyancy (F_b_), and magnetic forces (F_mag_) act on them. When cells get closer the equilibrium position, velocity of the cells and thus F_d_ and F_i_ become smaller and cells are levitated at the position where, F_mag_ and F_b_ equilibrate in opposite directions^38^. In this context, our magnetic levitation platform consists of two high grade (N52) neodymium (NdFeB) magnet (50 mm length, 2 mm width, and 5 mm height, Supermagnete) positioned at 1,5 mm distance with same poles facing each other, a micro-capillary channel (1 mm × 1 mm square cross-section, 50-mm length, Vitrocom) between two magnets and mirrors (12,7 × 12,7 × 3,2 mm, Thorlabs) at 45° for real-time inverted microscope imaging (Fig. 1a). The components of the magnetic levitation device are held together with photoreactive resin (Clear v2 FLGPCL02) printed using 3D printer (Formlabs Form 2).

### Cell Culture

D1 ORL UVA, D1 ORL UVA^eGFP^ (bone marrow stem cell line)^57^ and MDA-MB-231^dsRed^ cells were cultured in DMEM (Gibco) supplemented with 10% fetal bovine serum (FBS) and 1% penicillin/streptomycin. The cells were grown in a humidified 37 °C incubator with 5% CO_2_. The growth medium was changed every other day and the cells were passaged every four to six days.

### Short-term levitation of cells

D1 ORL UVA cells were thawed and centrifuged at 125 × g for 5 min and supernatant was discarded. The cells were resuspended to 10^5^ cells/ml in the culture medium with different Gd-based solutions; Gd-BT-DO3A (Gadavist^®^, Bayer), Gd-DTPA (Magnevist^®^, Bayer), Gd-DTPA-BMA (Omniscan^TM^, GE Healthcare), Gd-DOTA (Dotarem^®^,Guerbet) and Gd-BOPTA (Multihance^®^, Bracco) at variable concentrations of Gd^3+^ (0, 10, 25, 50, 100 and 200 mM), approximately 50 µl of cell suspension was loaded into the micro-capillary channel and the channel was sealed with Critoseal. The cells were levitated in the magnetic levitation device for 10 min and imaged every 1 min under the inverted microscope (Olympus IX-83). Levitation heights of cells (distance of cells from the bottom surface of micro-capillary channel) were measured with ImageJ Fiji software by performing threshold and particle analysis. In order to determine when cells reached to the equilibrium at a specific levitation height in different paramagnetic solutions, the time point that cells were approaching ±5% of the levitation height reached at 10 min was considered as equilibrium time. The levitation heights of D1 ORL UVA^eGFP^ and MDA-MB-231^dsRed^ cells in the medium containing 100 mM Gd-BT-DO3A were also measured by the same method.

### Cell viability assay

D1 ORL UVA cells were seeded at a starting concentration of 10^4^ cells/well in a 96-well plate and cultured for 48 h. The cells were exposed to different Gd-based solutions (Gd-BT-DO3A, Gd-DTPA, Gd-DTPA-BMA, Gd-DOTA and Gd-BOPTA) at variable concentrations of Gd^3+^ (0, 10, 25, 50, 100 and 200 mM) and cell viability was measured every 24 h for 3 d with thiazolyl blue tetrazolium bromide (MTT) assay. 0.5 mg/ml of MTT reagent (Amresco) was added to each well and the plates were incubated at 37 °C for 3 h in the dark. The media was removed, 100 μl of DMSO was subsequently added to each well and colorimetric measurements were performed at 570 nm with a reference wavelength of 690 nm (Thermo Scientific Multiskan Go). Furthermore, to evaluate whether stock concentration differences between commercial products (Gadavist^®^: 1000 mM; Magnevist^®^, Omniscan^®^, Dotarem^®^ and Multihance^®^: 500 mM) had an effect on cell viability, D1 ORL UVA cells were exposed to the medium containing phosphate buffer solution (PBS), instead of the contrast agent, (0, 10, 20, 30, 40 and 50%; v/v) for 24, 48 and 72 h and cell viability was measured by MTT assay. 20% and 40% PBS containing medium represent the dilution at the highest tested agent concentration (200 mM) of Gd-BT-DO3A and other contrast agents, respectively.

### Live/Dead Assay

D1 ORL UVA cells were seeded at a starting concentration of 4 × 10^4^ cells/well in a 24-well plate and cultured for 48 h. The cells were exposed for 72 h to Gd-BT-DO3A and Gd-DTPA-BMA at variable concentrations of Gd^3+^ (0, 10, 25, 50, 100 and 200 mM). Cell viability was assessed by live/dead assay (calcein-AM/propidium iodide, Sigma Aldrich). The cells were stained for 15 min and imaged under the fluorescence microscope (Olympus IX-83). For the assessment of long-term effect, the viability of cells exposed to 100 mM Gd-BT-DO3A for 120 hours in 2D monolayer culture and 3D magnetic levitation cultures (5000 cells/channel) were also analyzed by the same method. For 3D clusters assembled during weightlessness, cells were both investigated as clusters and as dissociated single cells.

### Levitation of cells at various temperatures

D1 ORL UVA cells were centrifuged at 125 × g for 5 min and supernatant was discarded. The cells were resuspended to 10^5^ cells/ml in the culture medium containing Gd-BT-DO3A (50, 100 and 200 mM concentrations of Gd^3+^). The sample (≈50 µl) was loaded into the micro-capillary channel and the channel was sealed. The magnetic levitation system was then placed within a microscope observation chamber in which temperature could be adjusted. The cells were levitated at 28, 32 and 36 °C, allowed to reach equilibrium (≈3 min) at each temperature and imaged under the inverted microscope (Observer Z1, Zeiss). Levitation heights of cells (distance of cells from the bottom surface of channel) were measured with ImageJ Fiji software and normalized to levitation heights of cells with magnetic levitation with 50 mM Gd^3+^ at 28 °C.

### Real-time cell assembly with magnetic levitation

D1 ORL UVA cells were detached by 0.25% trypsin, centrifuged at 125 × g for 5 min and medium was removed. The cells were resuspended to 10^5^ cells/ml in the culture medium with 100 mM Gd-BT-DO3A, cell suspension (≈50 µl) was loaded into the channel and the channel was sealed. The cells were levitated in the magnetic levitation device for 10 h and imaged every 15 min under the inverted microscope (Olympus IX-83) in a tiled acquisition manner to obtain images of the entire viewable area of the channel.

### Assembly of 3D cellular clusters with magnetic levitation

D1 ORL UVA cells (10^5^ cells/ml) were firstly levitated with 50 mM Gd-BT-DO3A, which appeared to be advantageous in terms of cell viability and sufficient levitation height of cells, and cultured in a humidified 37 °C incubator with 5% CO_2_ over 72 h. However, due to the fact that some cell spheres collapsed and proliferated on the ground of the micro-capillary channel, the concentration of Gd^3+^ was increased to 100 mM for long-term culture of cells with magnetic levitation. Briefly, after trypsinization, D1 ORL UVA^eGFP^ cells were harvested and resuspended to 10^5^ cells/ml (5000 cells/capillary), 10^6^ cells/ml (50000 cells/capillary) and 10^7^ cells/ml (500000 cells/capillary) in the culture medium with 100 mM Gd-BT-DO3A. The samples (≈50 µl) were loaded into the channel and the channel was sealed. The cells were cultured with magnetic levitation for 48 h at 37 °C and 5% CO_2_ in a humidified atmosphere and imaged every 24 h under the inverted microscope (Olympus IX-83). The long self-assembled clusters (>2.07 mm) were imaged in a tiled manner to obtain images of the whole cluster. When the reflection was distorting the image on the channel, these were removed manually. All geometric features of the self-assembled clusters were quantified with ImageJ Fiji. Threshold and particle analysis were performed to measure total area (A), perimeter (P) and position of the center of mass. Elongation was calculated the following equation: Elongation = P^2^/(4π × A). To quantitate positions of the clusters, the image of the same area, where the cluster was imaged, was captured by focusing on the magnets for each cluster and the position of the center of mass between magnets was determined by considering the top point of the bottom magnet = 0, the bottom point of the top magnet = 1. To calculate average thickness of the clusters, grid lines were added on the image to sample 5 points along the cluster (on the x-axis) at equal distance, the thickness values corresponding to these lines were measured ImageJ Fiji software using the “straight line” tool and averaged. The lengths of the clusters were measured as cluster’s distance of the starting and ending points on the x-axis. The geometric features of MDA-MB-231^dsRed^ clusters (5000 cells/channel) were also measured by the same method.

### Coculture assembly with magnetic levitation

Coculture assembly of D1 ORL UVA^eGFP^ and MDA-MB-231^dsRed^ cells with magnetic levitation (100 mM Gd-BT-DO3A) was performed using different cell loading strategies; L1, L2 and L3. In L1, D1 ORL UVA^eGFP^ and MDA-MB-231^dsRed^ cells (total ≈5000 or 50000 cells in ≈50 µl with 1:1 cell ratio) were loaded into the magnetic levitation system and cultured with magnetic levitation for 28 h. In L2, D1 ORL UVA^eGFP^ cells (total ≈2500 or 25000 cells in ≈25 µl) were cultured with magnetic for 24 h for single type cellular assembly. For coculture assembly, MDA-MB-231^dsRed^ cells (total ≈2500 or 25000 cells in ≈25 µl) were then added into the system and cultured for 4 h. In L3, MDA-MB-231^dsRed^ cells were cultured with magnetic levitation for 24 h, D1 ORL UVA^eGFP^ cells were added into the system and cultured for 4 h using the same cell numbers and medium volumes as the L2 experiment. The cells in the magnetic levitation system were cultured in a humidified 37 °C incubator with 5% CO_2_. The cellular clusters were imaged with the confocal (Leica DMi8) and fluorescence microscopy (Olympus IX-83).

### Statistical analysis

All experiments were repeated at least three times. Data are presented as mean ± standard deviation (SD). Coefficient of variation (CV%) was used as standard deviation/mean to reflect variability within and between experiments. Statistical significance was determined by Student’s t-test (two-tail) or two-way analysis of variance (ANOVA) with Sidak post hoc correction, through GraphPad Prism version 6.0 (GraphPad Software). P < 0.05 was considered statistically significant.

## Electronic supplementary material

Supplementary Information

Supplementary Video 1

Supplementary Video 2
