# Supplementary material for: Biofabrication of in situ Self Assembled 3D Cell Cultures in a Weightlessness Environment Generated using Magnetic Levitation
Source: Sci Rep. 2018 May 8;8:7239. doi: 10.1038/s41598-018-25718-9 (PMC5940762; doi:10.1038/s41598-018-25718-9)
Supplement: Supplementary file 1 — Supplementary Information [file 41598_2018_25718_MOESM1_ESM.pdf]

Biofabrication of in situ Self Assembled 3D Cell Cultures in a Weightlessness Environment Generated using Magnetic Levitation

Muge Anil-Inevi [1], Sena Yaman[1], Ahu Arslan Yildiz[1], Gulistan Mese [2], Ozden Yalcin-Ozuysal[2], H. Cumhur Tekin [1,*], Engin Ozcivici [1,*]

[1]*Department of Bioengineering*

[2]*Department of Molecular Biology and Genetics*

**equal correspondence*

Izmir Institute of Technology

Urla, Izmir, Turkey

*** Correspondence to:**

H. Cumhur Tekin, Ph.D.
Department of Bioengineering
Izmir Institute of Technology
Urla, Izmir, 35430
Turkey
Phone: + 90 232 750 7388
Fax: + 90 232 750 7603
e-mail: cumhurtekin@iyte.edu.tr

Engin Ozcivici, Ph.D.
Department of Bioengineering
Izmir Institute of Technology
Urla, Izmir, 35430
Turkey
Phone: + 90 232 750 7385
Fax: + 90 232 750 7603
e-mail: enginozcivici@iyte.edu.tr

Supplementary Information

Magnetic force ($\mathbf{F_{mag}}$) exerted on cells inside a magnetic field is given by Eq.1 [1]:

$$\mathbf{F_{mag}} = \frac{V.\Delta\chi}{\mu_o}(\mathbf{B}.\nabla)\mathbf{B} \qquad \text{(Eq. 1)}$$

where V is the volume of the cell, $\Delta\chi$ is the (volumetric) magnetic susceptibility difference between cell and paramagnetic medium ($\chi_c - \chi_m$), μ_o is the permeability of free space (1.257×10^{-6} kg·m·A^{-2}·s^{-2}), **B** is the magnetic induction (in Tesla, T), and ∇ is the del operator.

The cells that are located between two opposing magnets levitate at an equilibrium position where magnetic ($\mathbf{F_{mag}}$) and buoyancy force ($\mathbf{F_b}$) balance each other:

$$\mathbf{F_{mag}} + \mathbf{F_b} = 0 \qquad \text{(Eq. 2)}$$

Buoyancy force ($\mathbf{F_b}$) exerted on cells is defined by Eq. 3:

$$\mathbf{F_b} = V\Delta\rho g \qquad \text{(Eq. 3)}$$

where $\Delta\rho$ is the density difference between cell and paramagnetic medium ($\rho_c - \rho_m$) and g is the gravitational acceleration (9.8 m· s^{-2}).

Levitation equation can be stated using Eq. 1, Eq. 2 and Eq. 3 as follows:

$$\frac{V.(\chi_p - \chi_m)}{\mu_0}(\mathbf{B}.\nabla)\mathbf{B} + V.(\rho_c - \rho_m)\,\mathbf{g} = 0 \qquad \text{(Eq. 4)}$$

As magnetic susceptibility of the cells (χ_c) are negligible compared to the magnetic susceptibility of paramagnetic medium (χ_m) [2], susceptibility difference term ($\chi_c - \chi_m$) becomes negative, therefore the cells tend to move towards the location of minimum magnetic induction strength.

Magnetic induction (**B**) in our magnetic levitation platform using two opposing magnets was simulated by finite element method (FEM) (Supplementary Fig. 1). Residual induction value (Br) was taken as 1.42 T according to the product specifications (N52 Grade, Neodymium magnets, Supermagnete.de).

According to the simulation results, the magnetic induction strength reaches to its lowest value through the middle of the channel for both y and z-axes (Supplementary Fig. 1). Since $\mathbf{F_{mag}}$ is the only force exerted on cells along z-axis, they will be focused near the centerline of the capillary channel. Along y-axis, the cells will begin to levitate at the location where $\mathbf{F_{mag}}$ is balanced by $\mathbf{F_b}$ while they are moving towards the location of minimum magnetic induction (Fig. 1a).

References:

1 Pamme, N. Magnetism and microfluidics. *Lab on a Chip* **6**, 24-38 (2006).

2 Durmus, N. G. *et al.* Magnetic levitation of single cells. *Proceedings of the National Academy of Sciences* **112**, E3661-E3668 (2015).

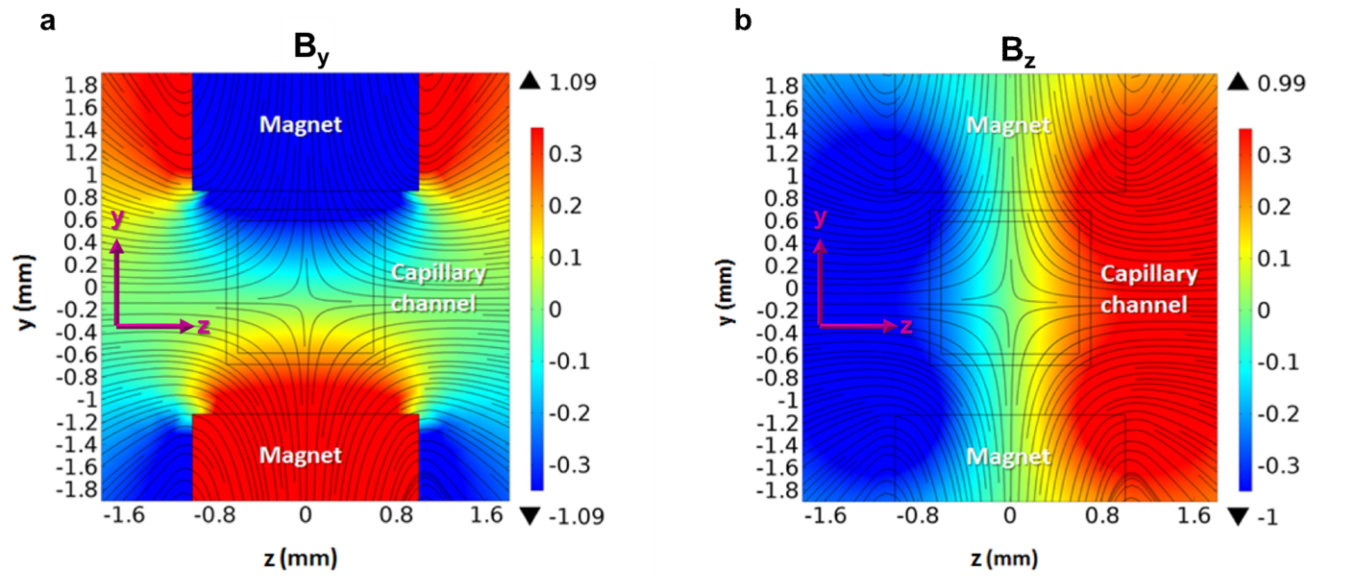

Supplementary Fig. 1. Simulation of (**a**) y component ($\mathbf{B_y}$) and (**b**) z component ($\mathbf{B_z}$) of magnetic induction (B) between two opposing magnets via Finite Element Methodology. Streamlines on the images represent total magnetic induction ($\mathbf{B_y+B_z}$).

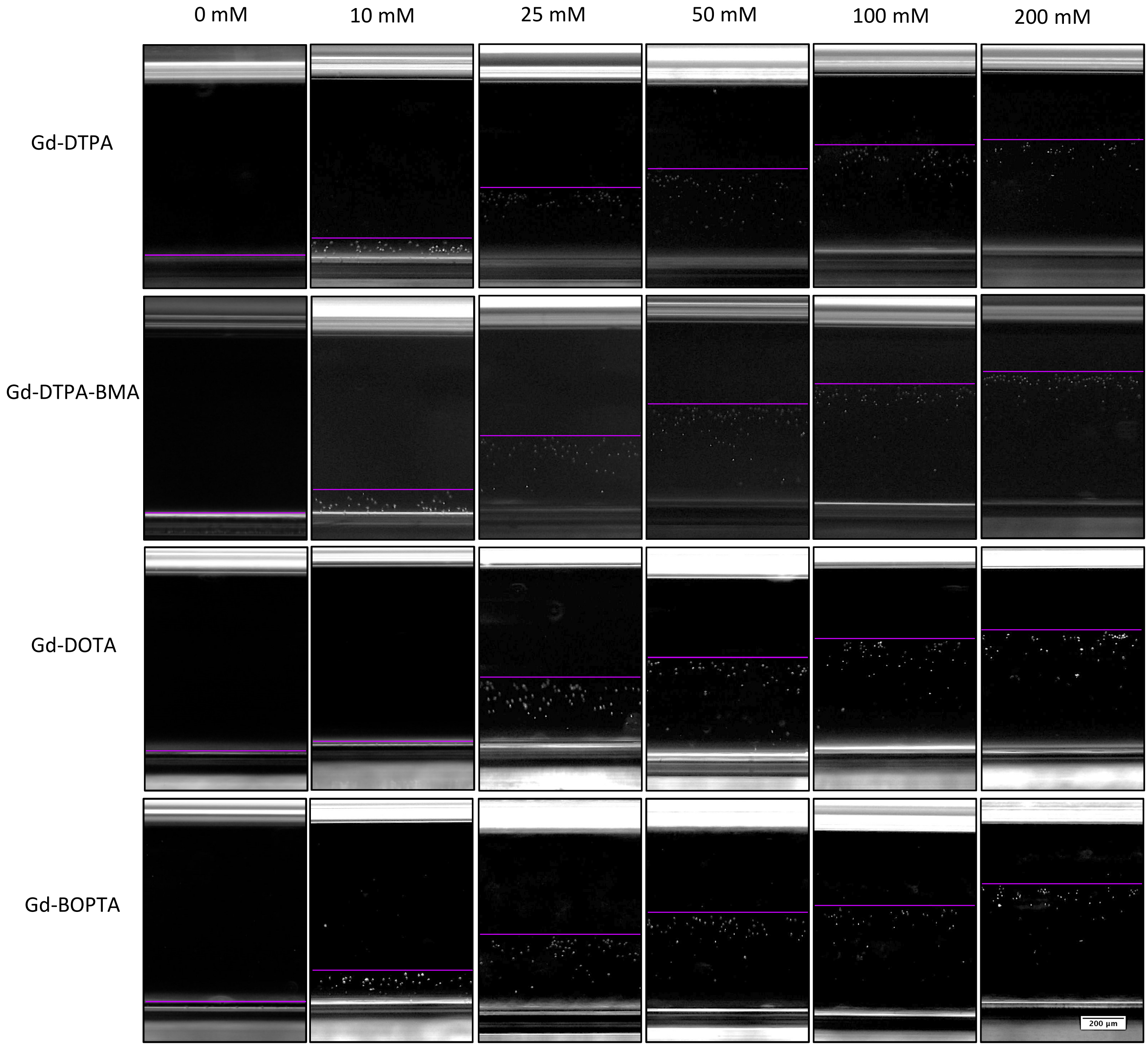

Supplementary Fig. 2 Micrographs of levitated D1 ORL UVA cells after 10 min of levitation in different Gd-based solutions (Gd-DTPA, Gd-DTPA-BMA, Gd-DOTA and Gd-BOPTA) at variable concentrations (0, 10, 25, 50, 100 and 200 mM). The lines show the upper level of the levitated cell population. Scale bar: 200 µm.

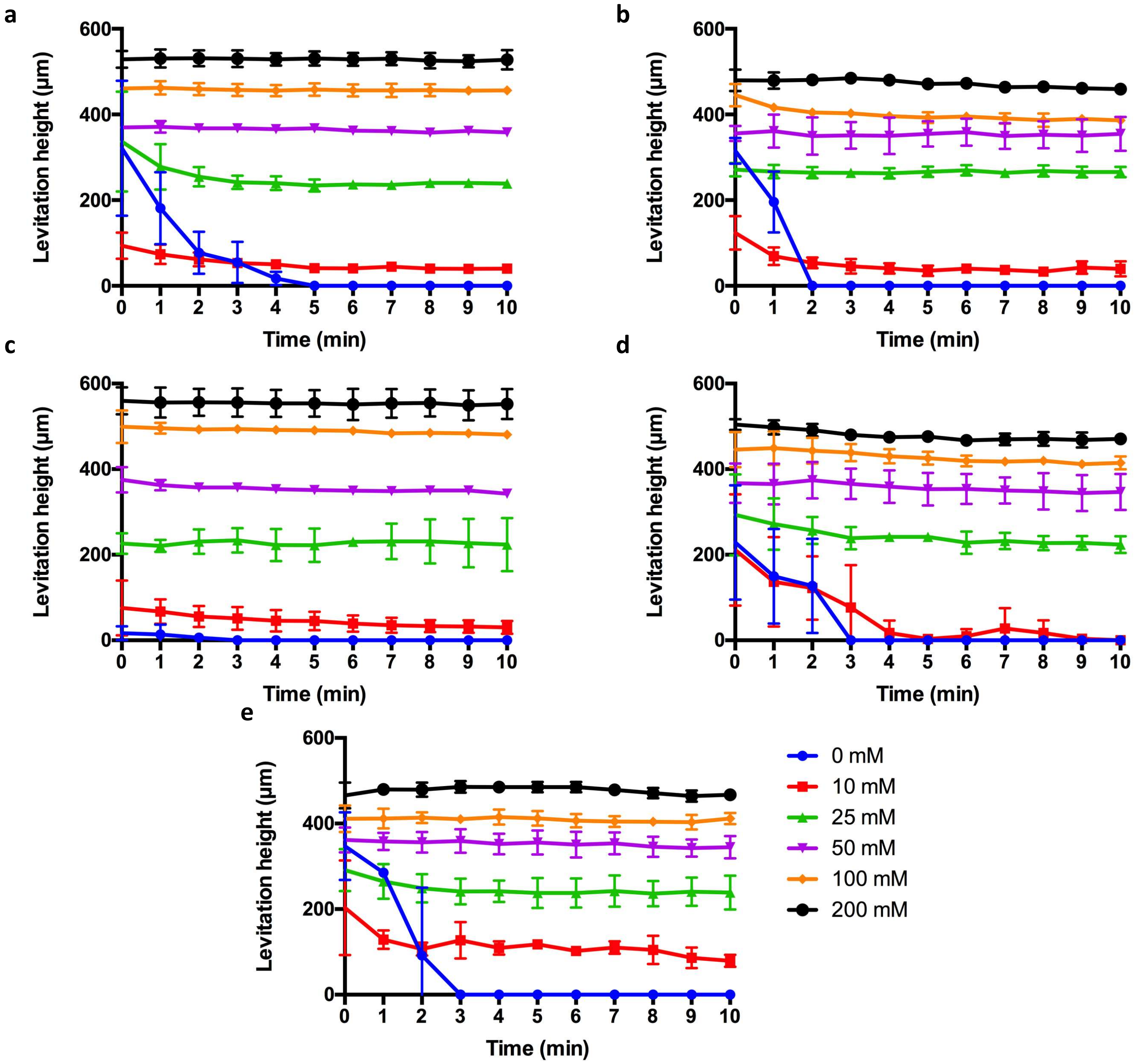

Supplementary Fig. 3 Time-dependent levitation heights of D1 ORL UVA cells (from bottom surface of capillary) levitated in different Gd-based solutions; (**a**) Gd-BT-DO3A, (**b**) Gd-DTPA, (**c**) Gd-DTPA-BMA, (**d**) Gd-DOTA and (**e**) Gd-BOPTA, at variable concentrations (0, 10, 25, 50, 100 and 200 mM) toward their equilibration point. Data are plotted as mean of replicates with error bars (± SD).

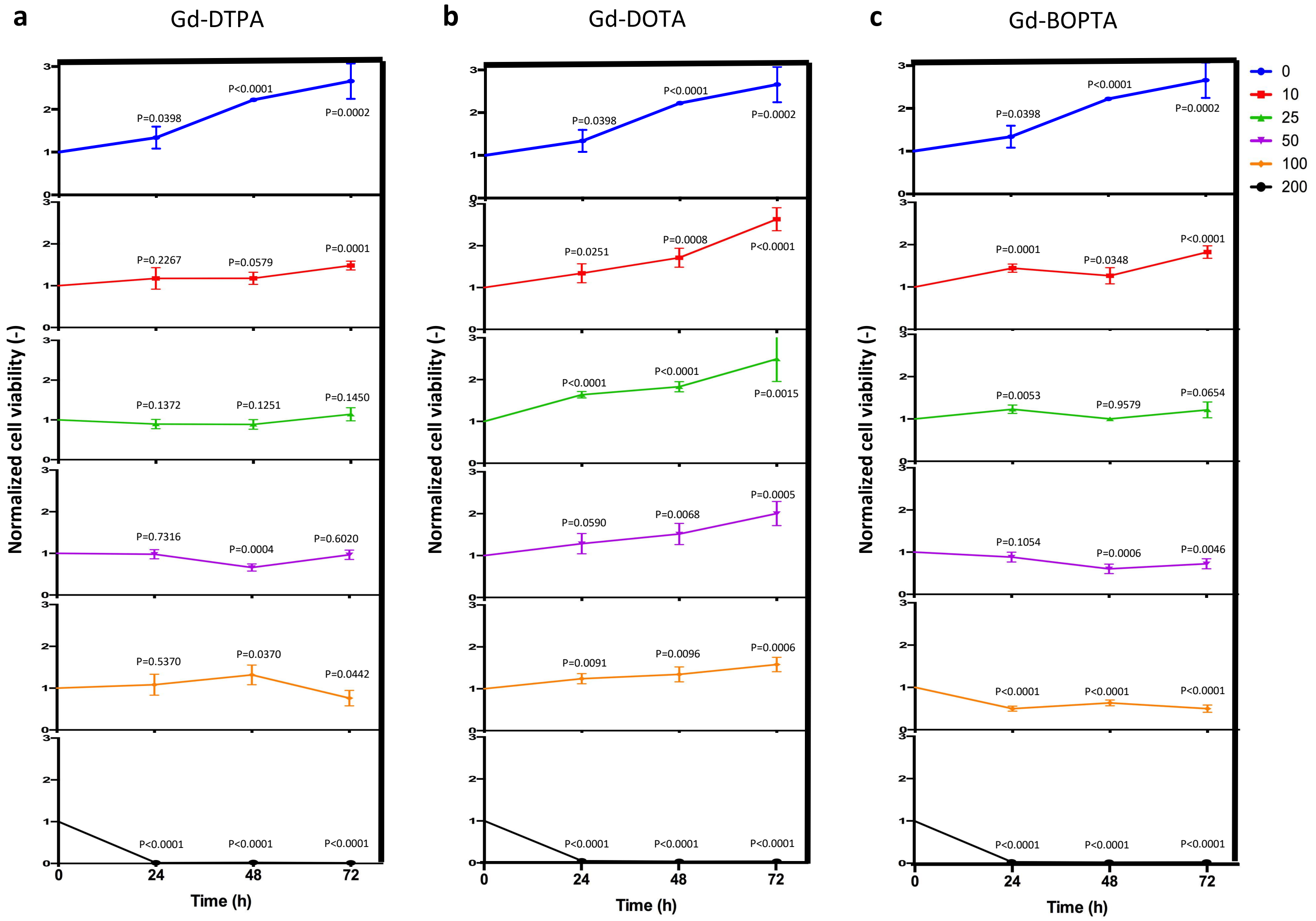

Supplementary Fig. 4 Cell viability for long-term culturing with (**a**) Gd-DTPA, (**b**) Gd-DOTA and (**c**) Gd-BOPTA at increasing concentrations (0, 10, 25, 50, 100 and 200 mM). Cell viability was determined with MTT assay. Data are plotted as mean of replicates with error bars (± SD). Groups were evaluated using the unpaired Student's t-test. Statistical significance was defined as P<0.05.

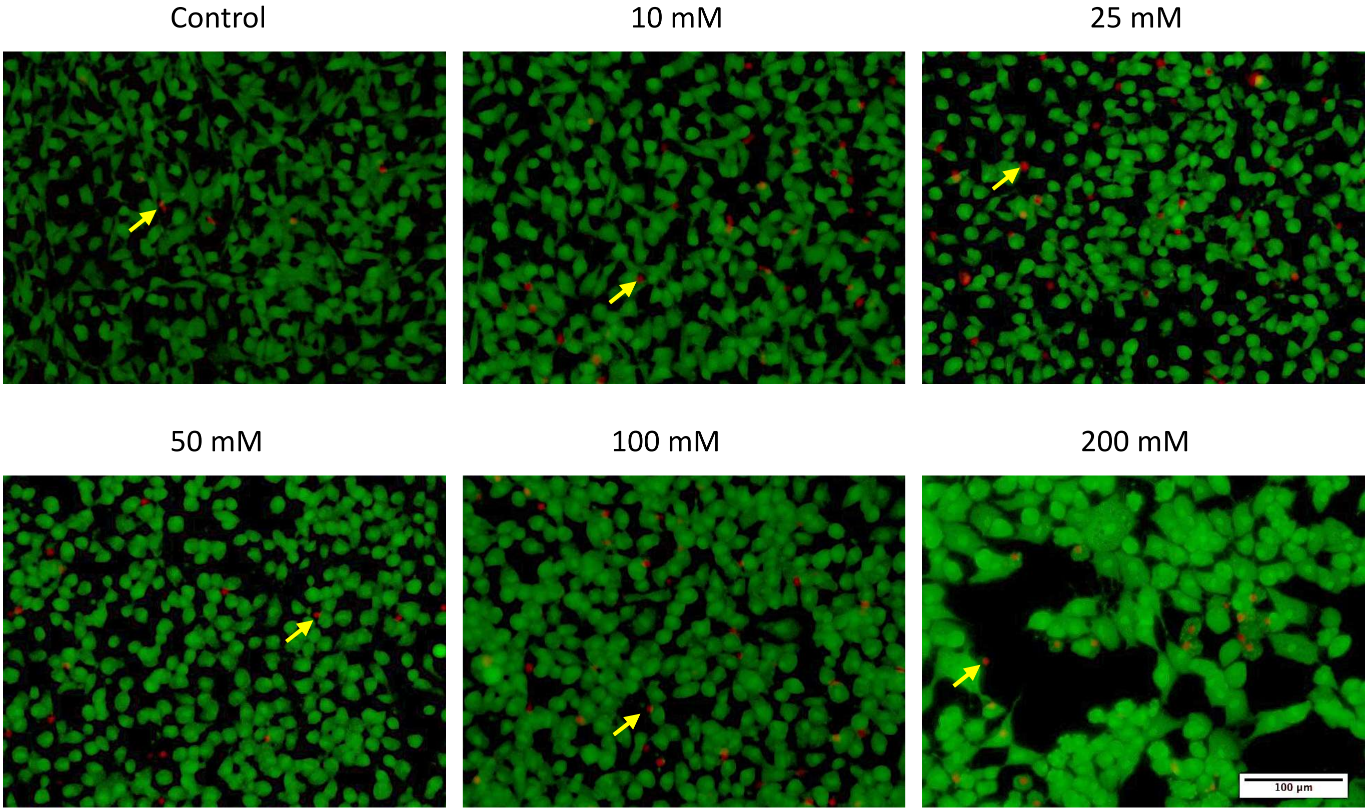

Supplementary Fig. 5 Zoomed-in depictions of the micrographs presented in **Fig. 2c**; D1 ORL UVA cells cultured for 72 h with 0, 10, 25, 50, 100 and 200 mM Gd-BT-DO3A (live: green, dead: red). The arrows indicate some of dead cells. Scale bar: 100 µm.

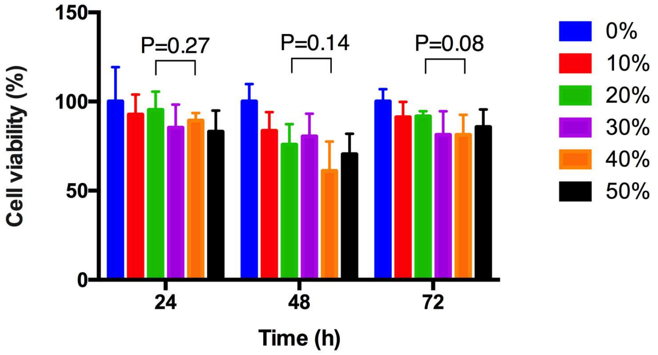

Supplementary Fig. 6 Cell viability of D1 ORL UVA cells cultured in 0, 10, 20, 30, 40 and 50% PBS (v/v) containing medium for 24, 48 and 72 h. Cell viability was determined with MTT assay. Data are plotted as mean of replicates with error bars (± SD). Groups were evaluated using the unpaired Student’s t-test. Statistical significance was defined as $P<0.05$.

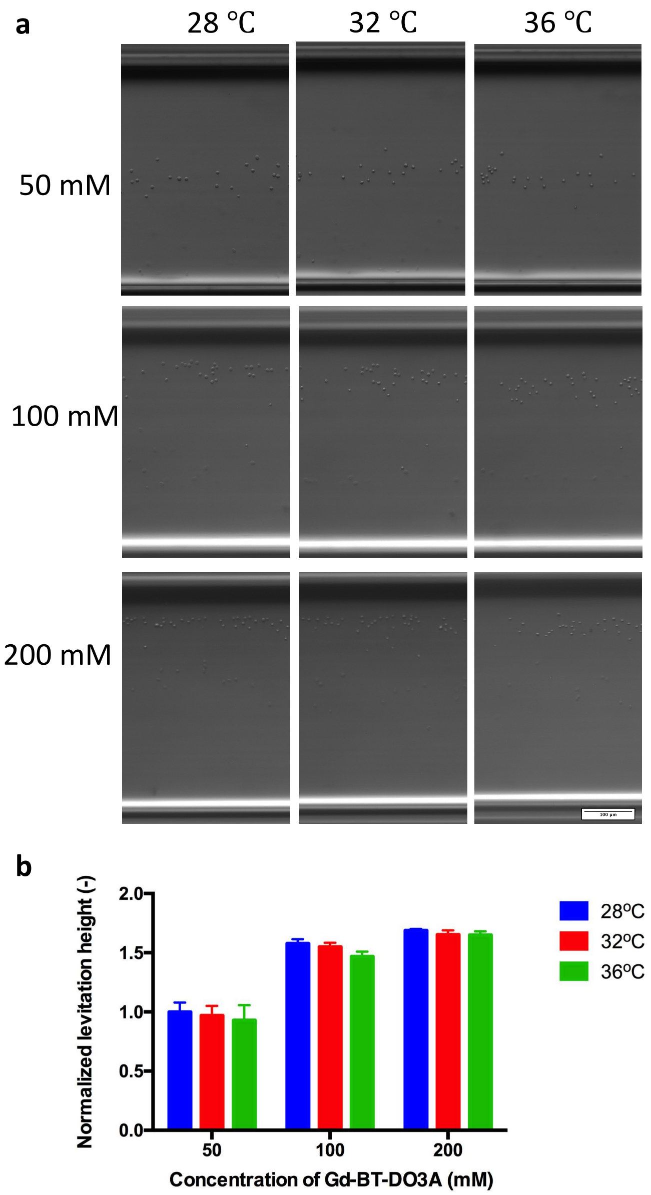

Supplementary Fig. 7 (**a**) Micrographs and (**b**) normalized levitation heights of levitated D1 ORL UVA cells at different Gd-BT-DO3A concentrations (50, 100 and 200 mM) after 3 min of levitation at 28, 32 or 36 °C. Data are plotted as mean of replicates with error bars (± SD) and statistically analyzed using a two-way ANOVA and Sidak posthoc test. Statistical significance was defined as $P<0.05$. Scale bar: 100 µm.

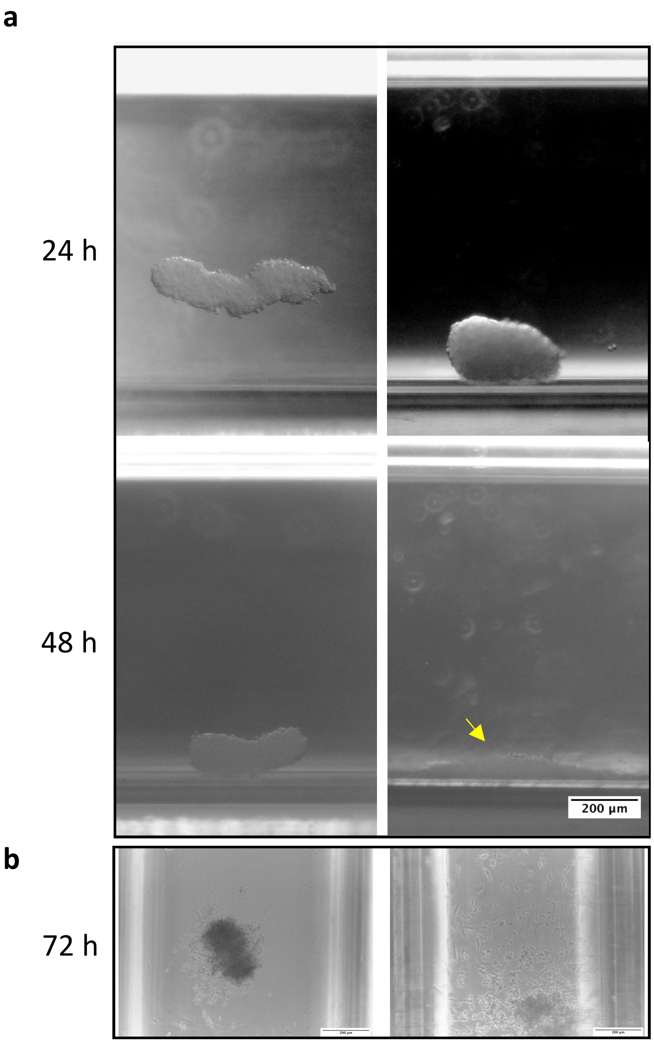

Supplementary Fig. 8 Micrographs of levitated D1 ORL UVA cells at 50 mM concentration of Gd-BT-DO3A after (**a**) 24 and 48 h of levitation in horizontal direction (across the capillary height) and (**b**) after 72 h in vertical direction (on the bottom of the capillary) (Scale bars: 200 µm). The arrow indicates a collapsed cellular cluster.

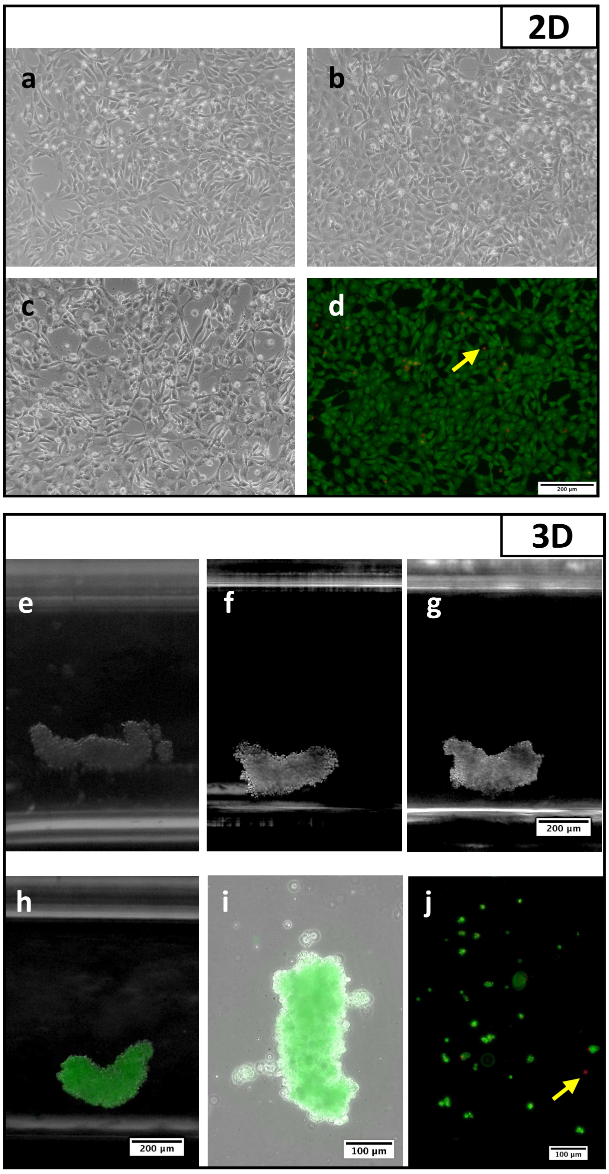

Supplementary Fig. 9 Micrographs of D1 ORL UVA cells exposed to 100 mM Gd-BT-DO3A in 2D culture for (**a**) 24, (**b**) 72 and (**c**) 120 h and live-dead image of cells after (**d**) 120 h. (Scale bar: 200 µm). Micrographs of levitated D1 ORL UVA cells at 100 mM Gd-BT-DO3A after (**e**) 24, (**f**) 72 and (**g**) 120 h (Scale bar: 200 µm). Calcein-AM staining of the D1 ORL UVA cluster assembled with magnetic levitation (100 mM, 120 h); (**h**) inside (Scale bar: 200 µm) and (**i**) outside the magnetic levitation device (Scale bar: 100 µm). **j**, Live/dead image of single cell suspension obtained from a cellular cluster assembled by magnetic levitation (100 mM, 120 h) (Scale bar: 100 µm). Cell viability was visualized by live-dead staining (Calcein/PI; live: green, dead: red). The arrows indicate dead cells.

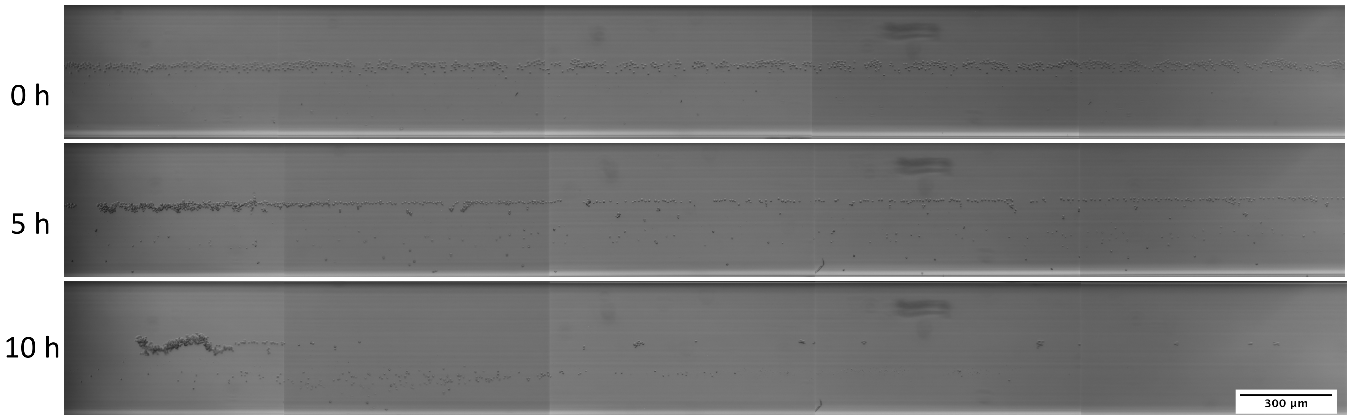

Supplementary Fig. 10 Micrographs D1 ORL UVA cells (total 5000 cell) levitated for 5 and 10 h (with 100 mM Gd-BT-DO3A). Scale bar: 300 µm.

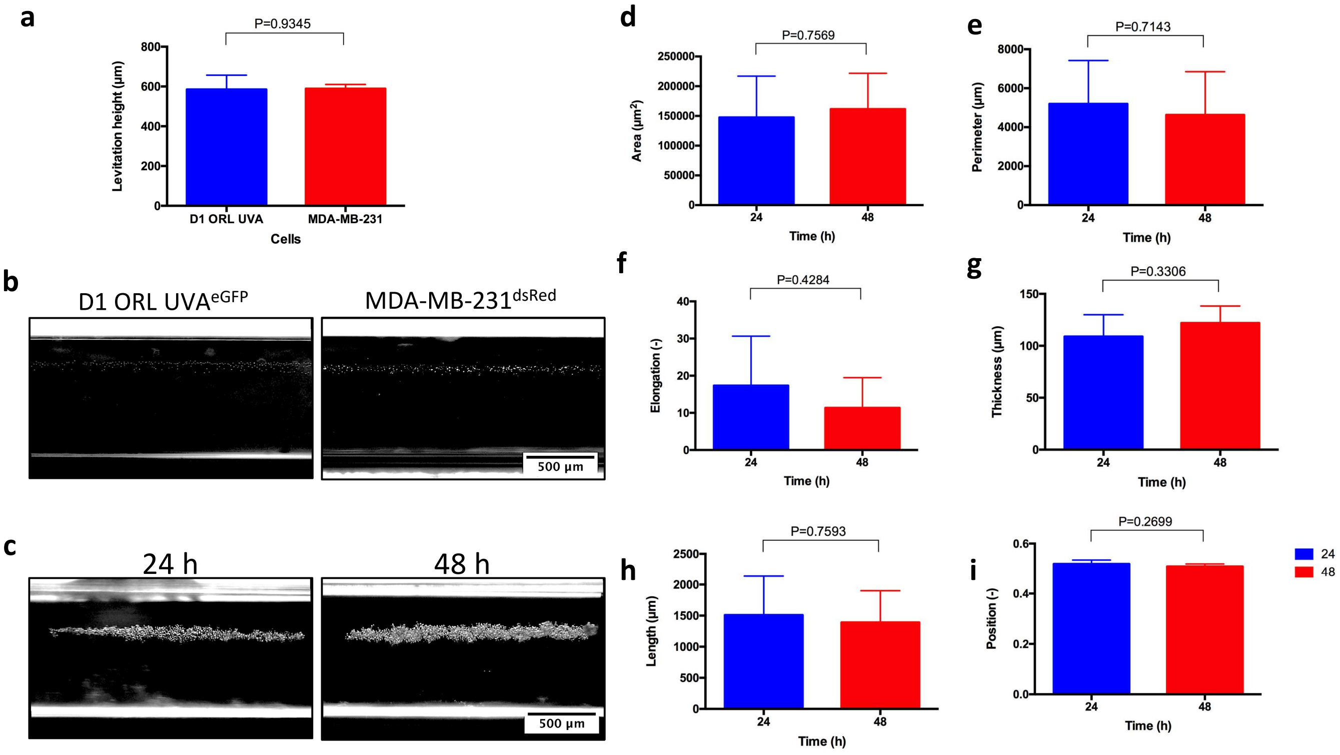

Supplementary Fig. 11 (**a**) Levitation heights (from bottom surface of capillary) and (**b**) microscopy images of D1 ORL UVA^{eGFP} cells and MDA-MB-231^{dsRed} cells after time of equilibrium at 100 mM concentration of Gd-BT-DO3A (total 5000 cells). (**c**) Microscopy images of self-assembled MDA-MB-231 clusters formed with magnetic levitation after 24 and 48 h of culture at 100 mM concentration of Gd-BT-DO3A (total 5000 cells). Scale bars: 500 µm. Quantitative description of MDA-MB-231^{dsRed} clusters formed for 24 or 48 h with magnetic levitation (100 mM Gd-BT-DO3A, total 5000 cells); (**d**) area, (**e**) perimeter, (**f**) elongation, (**g**) thickness and (**h**) length, and (**i**) position of the clusters between magnets (the top point of the bottom magnet:0, the bottom point of the top magnet:1). Data are plotted as mean of replicates with error bars (± SD). Groups were evaluated using the unpaired Student's t-test. Statistical significance was defined as $P<0.05$.

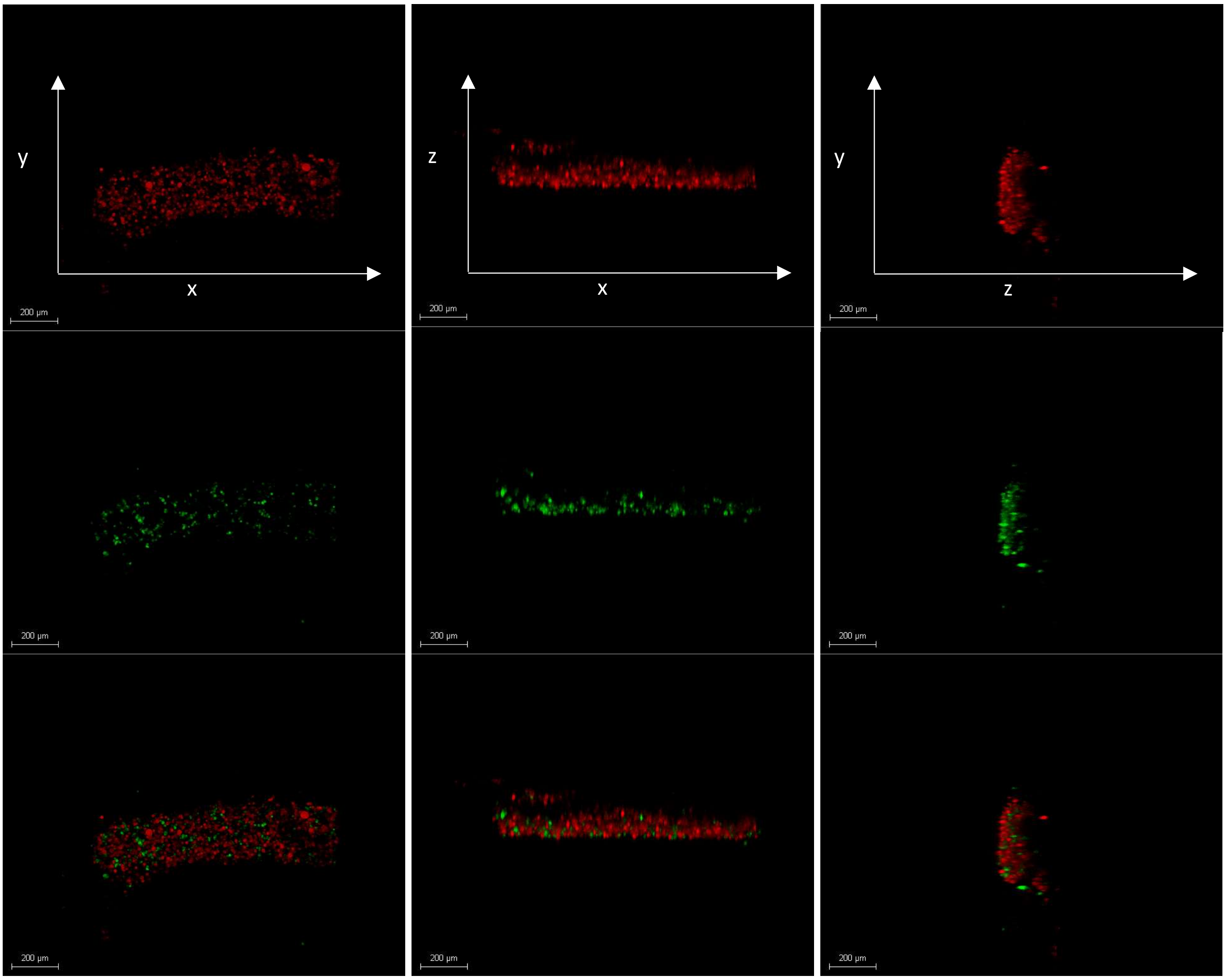

Supplementary Fig. 12 Micrographs showing cellular distribution of self-assembled clusters formed by L1 strategy. Green: D1 ORL UVA, Red: MDA-MB-231 (total 50000 cells with 1:1 cell ratio). Scale bar: 200 µm.

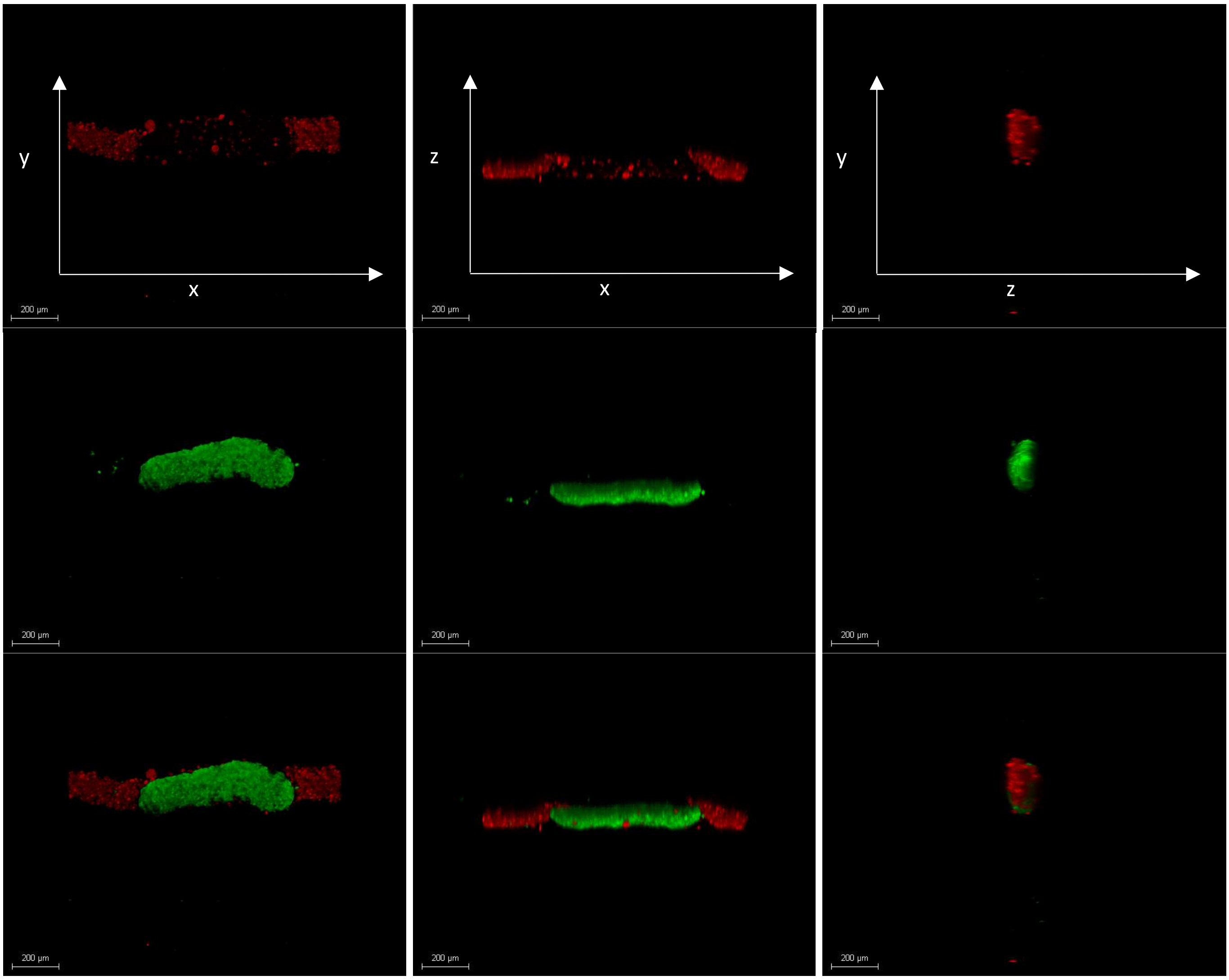

Supplementary Fig. 13 Micrographs showing cellular distribution of self-assembled clusters formed by L2 strategy. Green: D1 ORL UVA, Red: MDA-MB-231 (total 50000 cells with 1:1 cell ratio). Scale bar: 200 µm.

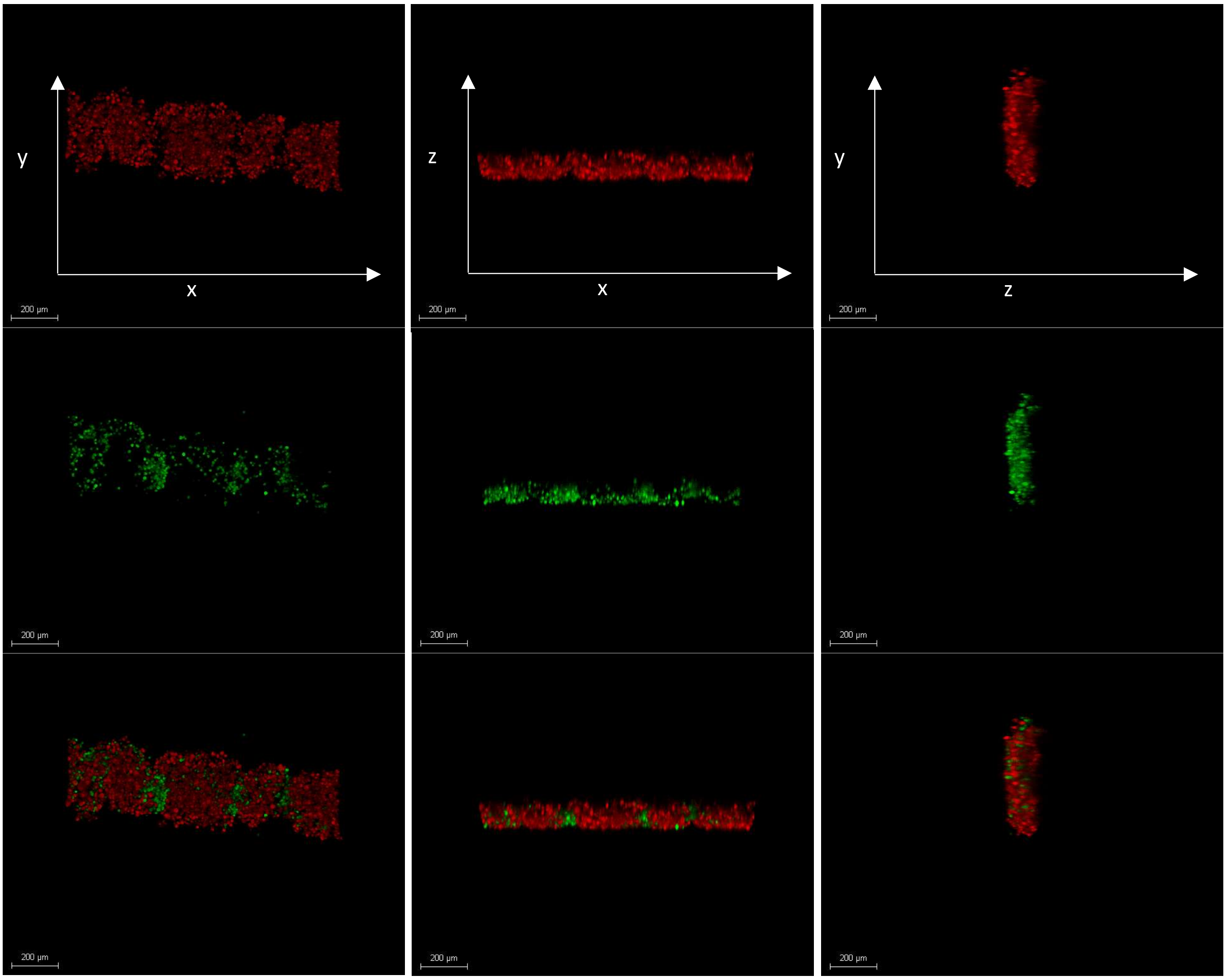

Supplementary Fig. 14 Micrographs showing cellular distribution of self-assembled clusters formed by L3 strategy. Green: D1 ORL UVA, Red: MDA-MB-231 (total 50000 cells with 1:1 cell ratio). Scale bar: 200 µm.
